# Autism spectrum disorder associated with 49,XYYYY: case report and review of the literature

**DOI:** 10.1186/s12881-017-0371-1

**Published:** 2017-01-31

**Authors:** Caroline Demily, Alice Poisson, Elodie Peyroux, Valérie Gatellier, Alain Nicolas, Caroline Rigard, Caroline Schluth-Bolard, Damien Sanlaville, Massimiliano Rossi

**Affiliations:** 1 GénoPsy, Centre for the Diagnosis and management of genetic psychiatric disorders, Centre Hospitalier le Vinatier and EDR-Psy team (CNRS & Lyon 1-Claude Bernard University), Lyon, France; 2 Unité Jouvet, Centre Hospitalier le Vinatier, Bron, France; 3 Centre de référence des anomalies du développement, Service de génétique, Hospices Civils de Lyon, & Centre de Recherche en Neurosciences de Lyon, Inserm U1028, UMR CNRS 5292, GENDEV Team, Lyon 1-Claude Bernard University, Bron, France

**Keywords:** XYYYY, Autism spectrum disorder, Social cognition, Neurocognition, Behavioural disorders

## Abstract

**Background:**

Sex chromosome aneuploidies occur in approximately one in 420 live births. The most frequent abnormalities are 45,X (Turner syndrome), 47,XXX (triple X), 47,XXY (Klinefelter syndrome), and 47,XYY. The prevalence of males with more than one extra sex chromosome (e.g. 48,XXYY or 48,XXXY) is less common. However, the literature provides little information about the cognitive and behavioural phenotype and the natural history of the disease. We report the clinical, neurocognitive, social cognitive and psychiatric characterization of a patient with 49,XYYYY syndrome.

**Case presentation:**

The patient presented with a complex phenotype including a particular cognitive profile with intellectual deficiency and autism spectrum disorder (ASD) with limited interests. Moreover, social anxiety disorder with selective mutism and separation anxiety disorder were observed (DSM-5 criteria, MINI Assessment).

**Conclusion:**

It is now admitted that 49,XYYYY has unique medical, neurodevelopmental and behavioural characteristics. Interestingly, ASD is more common in groups with Y chromosome aneuploidy. This clinical report suggests that understanding the cognitive and social functioning of these patients may provide new insights into possible therapeutic strategies, as cognitive remediation or social cognitive training.

## Background

Sex chromosome aneuploidies (SCA) occur in approximately one in 420 live births [1]. The most frequent abnormalities are 45,X (Turner syndrome), 47,XXX (triple X), 47,XXY (Klinefelter syndrome) and 47,XYY. The prevalence of males with more than one extra sex chromosome (e.g. 48,XXYY or 48,XXXY) is less common. To date, the psychiatric features of these syndromes have not been characterized precisely [2]. Nevertheless, it is important to understand the cognitive and social functioning of these patients since it may provide new insights into possible therapeutic strategies, such as cognitive remediation or social cognitive training.

48,XYYY and 49,XYYYY karyotypes are very rare: less than ten cases have been described for 48,XYYY and only three of them have been associated with a non-mosaic form. Eight cases with a majority of 49,XYYYY cells have been documented: all patients presented with mild to moderate intellectual disability (ID) and facial dysmorphic features such as hypertelorism, low-set ears and micrognathia. Clinodactyly and scoliosis were often associated. It is now recognized that 49,XYYYY has unique medical, neurodevelopmental, and behavioural characteristics. However, the literature provides little information about the cognitive and behavioural phenotype and the natural history of the disease [3].

We report for the first time, the clinical, neurocognitive, social cognitive and psychiatric characterization of a patient with 49,XYYYY syndrome. The dysmorphic features of the patient have been previously reported [4]. This patient presented with a complex phenotype including a distinct cognitive profile and autism spectrum disorder (ASD).

## Case presentation

The patient was the third child of healthy non-consanguineous parents. Pregnancy and delivery were normal. He was born at term with normal measurements. He presented with general developmental delay and dysmorphic features. General learning difficulties were also observed. He started walking at the age of 5 years old but had never walked on all fours. He presented major difficulties in acquiring and using language (first words: 4 years-old and first sentences: 6 years-old), and limited effective communication. He presented an immature language, with the use of jargon, pronoun reversal, prosodic disorders and monotonous tone. Language comprehension was delayed and the functional use of language impaired. The patient had difficulties with comprehension of humor, figurative expressions and jokes. He underwent consistent speech-language therapy from late infancy to adulthood, and specialized training.

Standard karyotype and fluorescence in situ hybridization (FISH) study using centromeric probes for X and Y chromosomes were performed at the age of 8 years old on blood sample and buccal swab. They showed 49,XYYYY karyotype in all the examined cells, namely 100 lymphocytes and 150 buccal cells [4].

The patient was 34 years old when he was referred to our Center. His height was 193.5 cm (+3SD), his weight was 104.8 kg (+4SD) and his OFC was 60 cm (+3SD). Body Mass Index was increased at 28,2 (20 < normal values < 25) with a central adiposity. Unfortunately, histological growth records were not available for the patient.

**Fig. 1:**
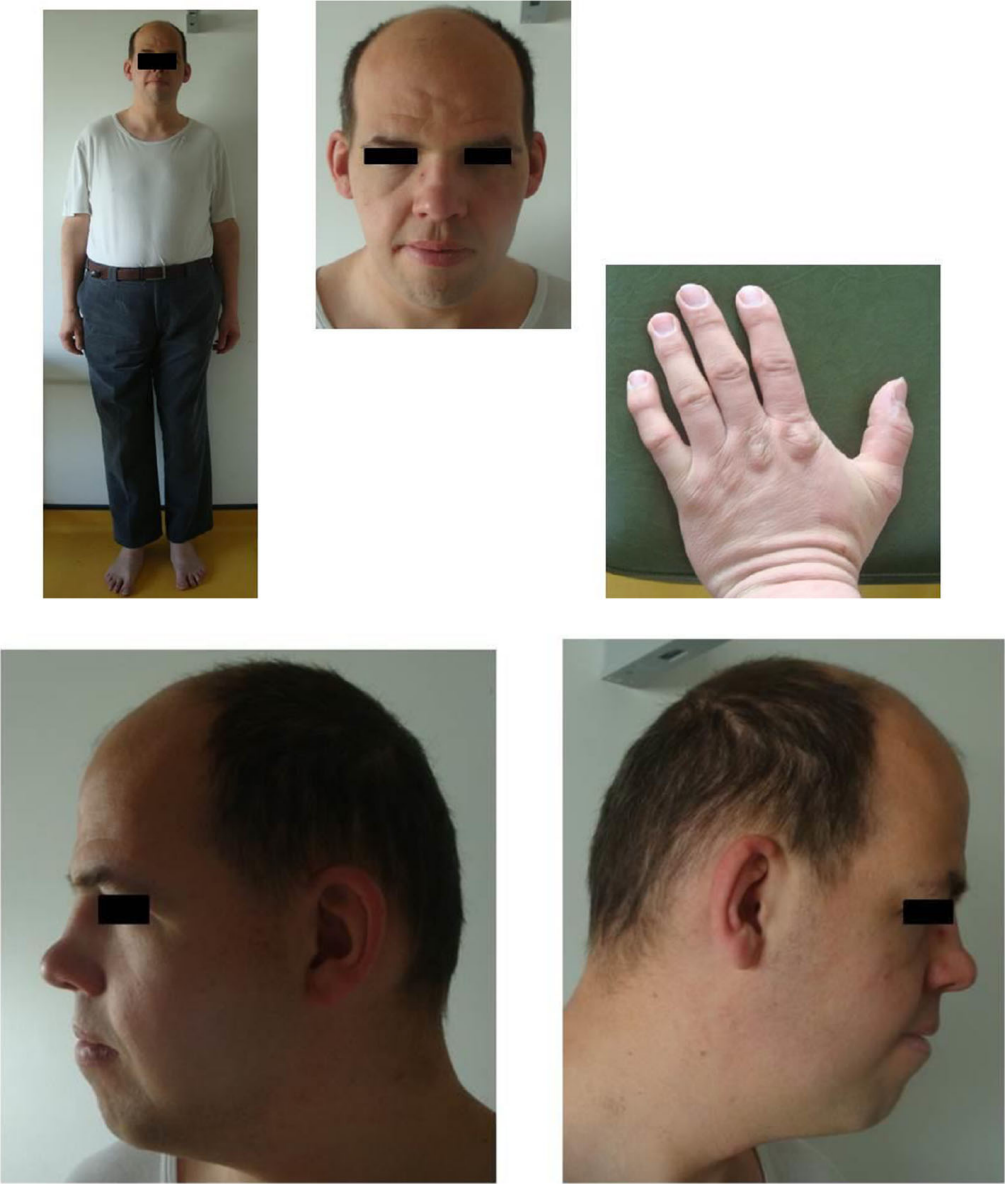
Dysmorphic features

Clinical examination showed tall stature, macrocephaly, turricephaly and brachycephaly, high forehead, long face, oedematous eyelids, narrow palpebral fissures, bulbous nasal tip, thick lips, thick helix, multiple caries, and mild clinodactyly of the fifth fingers (Fig. 1).

He had essential tremor treated with propranolol, severe sleep disorders (he fell asleep very frequently during the day), and diabetes treated with gliclazide and metformin.

Laboratory investigations showed normal blood cell count, TSH, iron and calcium levels and a normal liver, renal and gonadal functioning. Pelvic imaging findings were within the normal limits. For ethical reasons (no medical indication, lack of child project and behavioral disorders), spermatogenesis was not evaluated.

**Fig. 2:**
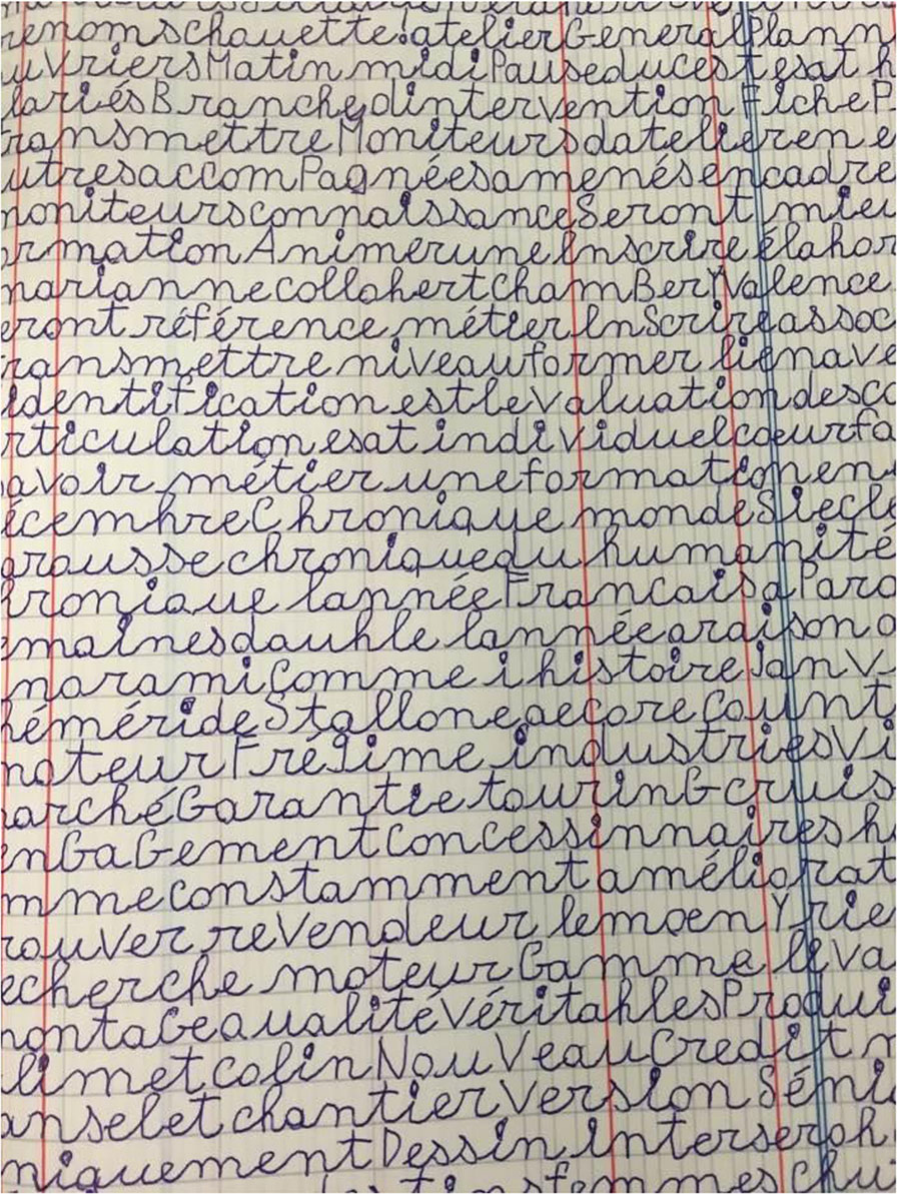
Repetitive writing

He lived with his father. His daily activities remained very limited and obsessive (Fig. 2). In case of frustration, the patient showed aggressive verbal behaviour and impulsivity.

## Psychological and functional assessments

### Cognitive abilities

The patient underwent neuropsychological assessment to define his cognitive profile. IQ measurement [5, 6] revealed a mild intellectual disability (total IQ = 59 [42–55] verbal IQ = 55; performance IQ = 63 – WAIS-III [6]). Difficulties were observed in almost all cognitive areas (Table 1). The patient did not exhibit behavioral or cognitive criteria for Attention Deficit Hyperactivity Disorder (ADHD).

**Table 1:** Cognitive profile

DOMAINS	TESTS	SCORES
Vocabulary	Peabody Picture Vocabulary	Raw score: 74
	Test – Revised [7]	Standard score: 41
Visual exploration and analysis	Position Discrimination –	Raw score: 7 (cut-off: 7)
	Visual Object and Space Perception Battery [29]	
	Number Location – VOSP [29]	Raw score: 0 (cut-off: 18)
	Picture Completion – WAIS III [5, 6]	Raw score: 15 Standard score: 4
Visual memory	Doors test [30]	Total raw score: 19/24
		Percentile: 25-50^th^
Attention	Alertness - TAP 2.3 [31]	With signal - Percentile: 8^th^
		Without signal - Percentile: 18^th^
Executive functions	Go/NoGo - TAP 2.3 [31]	Mistakes: 17
		Percentile: <1^th^
	Category fluency task – Grefex [32]	Raw score: 4
		Percentile: <5^th^

### Speech and language difficulties

Severe expressive language delay was observed (evaluated by a trained speech therapist [7]), with specific phonologic disorders including cognitive-linguistic disorders reflecting inaccurate or incomplete phonologic representations or inappropriate phonologic rules.

### Adaptive skills and behaviour

Adaptive skills were more limited than expected from IQ score. Median performance identified severe adaptive deficit compared to average. According to his father’s report on the Vineland Adaptive Behavior Scales [8], the patient had difficulties in the Communication (raw score: 70; standard score <20), Daily Living Skills (raw score: 88; standard score <20) and Socialization (raw score: 47; standard score <20) domains.

Social anxiety disorder with selective mutism and separation anxiety disorder were observed (DSM-5 criteria, MINI Assessment). ADOS (Autism Diagnostic Observation Schedule, [9]) was used to assess communication, social interaction abilities, creativity and the imaginative use of objects (Table 2).

**Table 2:** ASD symptoms

• No response to his name by 12 months of age • No pointing at objects to show interest by 14 months of age • Difficulties understanding other people’s feelings (Theory of Mind disability) • Delayed speech and language skills • Extreme reactivity to minor changes • Restrictive and repetitive interests and stereotypies • Lack of interest in sharing with others • Inappropriate and poor facial expressions • Misunderstanding of personal space boundaries • Avoidance or resistance to physical contact • Unusual emotional reactions	

The patient presented with

In the “communication” category, the patient developed fairly good productive language skills. He was able to produce short sentences and did not show any tendency to echolalia, even if some expressions or turns of phrase sometimes seemed stereotyped. His tone of voice was sometimes monotonous or, on the contrary, exaggerated or mannered. He was able to produce appropriate reports of routine events but needed to be asked questions. During the assessment, he did not provide any expected spontaneous information about his few interests. Reciprocal communication was limited, since he tended to follow his own idea rather than participate in the conversation. Consequently, his ability to request information was limited (ADOS communication score: 5; Autism Cut-Off: 3/Autism Spectrum Disorder Cut-Off: 2).

Nevertheless, the patient was able to use descriptive, conventional, instrumental and informative gestures to communicate, even if they were not always perfectly adapted to the context. He was also able convey emotions through gestures, although the same limitation was observed.

We observed poor and limited eye contact in reciprocal social interactions during the ADOS examination. Sometimes, he was able to display facial expressions to communicate an affect. Non-verbal communication associated with language was rare and very limited. He also had difficulty communicating his own affect and did not respond or react to the examiner’s emotional state (ADOS Social interaction score: 11; Autism Cut-Off: 6/ASD Cut-Off : 4) [9].

We noted that the patient had greater difficulties understanding his responsibility and his role in typical social relationships. In addition, he did not initiate any social contact even if he was able to respond in a limited way to all social situations.

Concerning imagination, he did not initiate any spontaneous creative actions or pretend plays, except when he was invited to do so, i.e. in situations that were purposely designed for it.

His behaviour did not reveal any unusual sensory interests, but the examiner noted motor mannerisms of hands and fingers, as well as scratching without self-harm behaviour. Restricted and repetitive interests were observed (Fig. 2), but the assessment did not highlight any rituals or compulsions. Finally, he did not present with hyperactive, aggressive or disruptive behaviour during the ADOS examination. Nevertheless, he showed symptoms of anxiety (tremors, fear of loosing control, more pronounced repetitive behaviors) throughout the ADOS assessment that were more evident when he had a difficult time completing the task.

### Sleep disorder

Two-night polysomnography was performed. We observed shortened (4 h 56 min and 6 h 17 min) and fragmented sleep duration (wake after sleep onset = 283 and 214 min). Only three sleep cycles were identified. Nevertheless, the percentages of slow-wave sleep (14.5 and 14%) and rapid eye movement sleep (19.8 and 19.5%) were in the normal range. No sleep apnoea syndrome was detected. Since the patient complained about excessive daytime sleepiness, we can hypothesize that he experienced reduced day/night contrast with equal distribution of sleep pressure between daytime and night-time.

## Cytogenetic analysis

Genetic analyses were performed after obtaining the patient’s signed informed consent, in accordance with French legislation. Such analyses are performed routinely and do not require specific approval by any ethical committee.

### Chromosome analysis

Blood karyotype (both GTG- and RHG banding) was performed in accordance with standard methods.

### Fluorescence In situ hybridization (FISH)

FISH was performed with DXZ1/DYZ3 probes, i.e. the centrometric probes for both X and Y chromosomes. One hundred mitoses and 200 nuclei were analysed.

## Results

Standard blood karyotype showed the presence of one X chromosome and four Y chromosomes in 24 out of 25 mitoses, and one mitosis with only one X chromosome. FISH confirmed the presence of two cell populations: one population with one signal for the X chromosome and four signals for the Y chromosome in 86 mitoses (86%) and 174 nuclei (87%); and one population with only one signal for the X chromosome in 14 mitoses (14%) and 26 nuclei (13%). To sum up, the patient had chromosomal mosaicism combining XYYYY population in about 85% of cells and monosomy X in about 15% of cells. Standard karyotype performed in the father was normal.

## Discussion and conclusion

Our observation reports ASD associated with XYYYY pentasomy and highlights two important points:

i) the patient with XYYYY pentasomy met criteria for ASD, and ASD has been found in other studies of males with Y chromosome polysomy,
ii) the importance of developing a psychiatric personalized evaluation using standardized assessment ASD measures in order to plan a “bottom up” treatment

i) *Y chromosome and ASD* (Table 3)

**Table 3:** Review of published case reports

	Age at diagnosis	Facial features	Skeletal abnomalities	Stature	Psychomotor developpement	Behavioral features	Testicular insufficiency	Cytogenetic analysis
Present case	8 years	Macrocephaly, turricephaly and brachycephaly, high forehead, long face, oedematous eyelids, narrow palpebral fissures, bulbous nasal tip, thick lips, thick helix	mild clinodactyly of the fifth fingers	Tall	Mild ID Speech delay	ASD Anxiety Sleep Disorders		Mosaicism 49, XYYYY (85%), 45,X0 (15%)
[23]	30 years	Proeminent forehead and supraorbital ridges	Radioulnar synososis clinodactyly of the fifth fingers	Normal Range	Severe ID	Violent behavior	Azoospermia	49, XYYYY
[24]	14 months	Low set ears Micrognatia Trigonocephaly Epicanthal folds Palate hight arched	Radio Ulnar synostosis Scoliosis brachyclinodactyly	Normal Range	Psychomotor retardation Speech delay	Impulsivity Low frustration threshold	Increased basal gonadotropins	49, XYYYY
[33]	6 years	Low set ears Bilateral « lop ears »	Turricephaly	Tall	Speech delay	Low frustration threshold Mild social interaction disorders Attention deficit		49, XYYYY
[25]	15 years	Bilateral Cataract Bradycardia	Clinodactyly of the fifth fingers	Normal Range	Psychomotor retardation		Absence of spermatogenesis	Mosaicism 45,X/49, XYYYY = 88%
[22]	8 days	Micrognatia Bulbous nasal tip Low set ears Palate hight arched	Radioulnar synostosis Clinodactyly scoliosis	Normal Range	ID hypotonia			Mosaicism 49, XYYYY (96,7%)
[20]	9 years		Joint laxity scoliosis	Short	Mild ID	Impulsivity		Structural rearrangement 45,X/47,X + 2 Iso dic Y
[17]	2 years	Transient Atrioseptal defect	Bilateral Radioulnar synostosis clinodactyly of the fifth fingers	Short	Short Mild ID Langage delay		Decreased testosterone	Structural rearrangement

The patient seemed to have specific social interaction and communication impairments that could not be explained by cognitive difficulties alone, and further evaluation showed that he met diagnostic criteria for ASD. Various studies have revealed a specific social functioning profile in males with SCA, suggesting vulnerability to autism, more severe in the post-natally ascertained boys [10]. In a study assessing the risk of ASD in 62 males with SCA (20 XXY, 22 XYY and 20 XXYY gonosomal systems), none of 47,XXY, 36% of 47,XYY, and 50% of 48,XXYY patients were diagnosed with ASD according to the SCQ and ADOS-G The severity of ASD was negatively correlated with verbal IQ and adaptive functioning in XYY and XXYY males. In this study, no aCGH or exome sequencing was performed, and a putative variant located elsewhere on the genome may explain such symptoms [11]. Our patient also had low IQ and very low adaptive functioning. This IQ/adaptive functioning gap has been previously reported in various studies.

Another study included 26 boys with 47,XYY, 82 boys with 47,XXY, and 50 controls (ages 4–15 years). Fifty per cent and 12% of the XYY and XXY groups, respectively, had scores >15 for autism screening from the Social Communication Questionnaire. For the boys with XXY, prenatal diagnosis was associated with fewer problem behaviors [12]. Concerning Social Responsiveness Scale scores, patients with XXY had lower (better) scores compared to XYY and XXYY patients, without significant differences between XYY and XXYY [13]. In a sample of children with XXX, XXXX, XXXXX, XYY, XXY, XXXY, and XXXXY and typically developing controls, Lee et al. [14] demonstrated a significant effect of Y-chromosome number on IQ and ASD symptomatology. Supernumerary Y-chromosomes were associated with impairments in both structural and pragmatic language.

In addition to core symptoms, an estimated 40% of children with ASD fulfill diagnostic criteria for an anxiety disorder and as many as 84% have impairing, subclinical anxiety symptoms [15]. Co-occurring anxiety can cause acute distress, amplify the core symptoms of ASD and trigger behavioral difficulties including tantrums, aggression and self-injury [16], as it was described in our case report. Despite the prevalence of anxiety in ASDs, the specificity remains unclear whether anxiety difficulties constitute a separate condition or align more closely with core ASD features. Boys with XYY did not report increased sentiments of anxiety or depression, compared with the general population [10]. To our knowledge, this specific issue has not been adressed in Y pentasomy.

There are few documented cases of male patients with XYYY and XYYYY syndromes. These syndromes result from Y-chromosome nondisjunction during spermatogonial mitosis associated with nondisjunction in meiosis. All descriptions in the literature have reported ID with behavioural disturbances, but the characterization of the psychiatric phenotype remains limited.

Tetrasomy Y is clinically exceptional, since only 11 cases have been described. These include seven cases without mosaicism, three cases with mosaicism (49,XYYYY > 50%), two cases with 48,XYYY >50%, and five cases with XXXY/XXXXY syndrome. All patients presented with mild to moderate ID, dysmorphic facial features and skeletal malformations ([3, 4, 17–25]).

Van den Berghe et al. [25] reported a case with comparable 45,X/49,XYYYY mosaicism. The patient presented with psychomotor deficiency and complete absence of spermatogenesis. The phenotypic characteristics were different, with middle-ranged size, bilateral cataract, facial asymmetry and cardiac abnormalities. Sirota et al. [24] reported a case with visual motor disorders, difficulties with coping strategies and lack of confidence. In the present case, our patient did not exhibit physical features classically observed in monosomy X (absence of pterygium colli, absence of lymphedema, skeletal abnormalities, renal or heart defects). However, developmental delays, nonverbal learning disabilities, and behavioral problems are possible in monosomy X, although these characteristics vary among affected individuals.

Interestingly, Margari et al. [2] reported ASD symptoms in approximately 23% of 47,XYY patients. Our study further supports the involvement of supernumerary Y chromosomes in the aetiology of ASD. To our knowledge however, no gene on the Y chromosome has been associated with ASD in the literature. This lack of data remains the main limitation of this observation.

The origin of the phenomenon deserves further investigation to determine the influence of biology and behaviour. Both ADHD and ASD are important clinical considerations in male patients with SCA, although ADHD was not observed in the patient. Interestingly, ASD is more common in groups with Y chromosome aneuploidy. Advances in genetics have substantially expanded knowledge of potential mechanisms that underlie these phenotypes. Indeed, a putative dose effect of sex chromosome genes on cognitive abilities is documented [26].

ii) *An appropriate treatment considering a “bottom up” approach.*

The major issue of the present case is comorbidity of Y pentasomy and ASD because unrecognized autistic spectrum disorder could have an impact on case management. ASD have different presentations depending on the genetic etiology. In that connexion, our case report provides evidence for social cognitive specific features in 49,XYYYY syndrome. The impact on social cognition of several kinds of interventions has been studied recently. In adults, satisfactory results for improving social cognition in ASD are those obtained by “bottom-up” approaches such as cognitive remediation therapy, social cognitive training and/or serious games [27]. Several new cognitive remediation strategies and programs are currently being developed. In our experience, attentional and social cognition deficits have a negative impact on adaptative and social competences and, as a result, on the ability to achieve a normal functioning. The improvement of attentional and social cognitive deficits thanks to a specific cognitive remediation program could have a positive impact on the behavior [28]. This approach would complete the reeducation methods already available. In conclusion, there is an urgent need for researchers to prioritise a better characterization of genotype/cognitive phenotype correlation to further advance therapeutic perspectives.

## Abbreviations

aCGH: Microarray-Based Comparative Genomic Hybridization
ADHD: Attention Deficit Hyperactivity Disorder
ADOS: Autism Diagnostic Observation Schedule
ASD: Autism Spectrum Disorder
DSM-5: Diagnostic and Statistical Manual – Fifth revision
FISH: Fluorescence In Situ Hybridization
ID: Intellectual Deficiency
SCA: Sex Chromosomal Aneuploidy
SCQ: Social Communication Questionnaire
SD: Standard Deviation
